# Immunomodulatory drugs: Oral and systemic adverse effects

**DOI:** 10.4317/medoral.19087

**Published:** 2013-08-29

**Authors:** Antonio Bascones-Martinez, Riikka Mattila, Rafael Gomez-Font, Jukka H. Meurman

**Affiliations:** 1 MD, DDS, PhD, Chairman of Oral Medicine and periodontology, Department of Medicine and Bucofacial Surgery, Dental School, Complutense University of Madrid, Plaza Ramón y Cajal 3, Ciudad Universitaria, 28040-Madrid, Spain; 2 MD, MSc, DDS, PhD, Specialist training, Department of Oral and Maxillofacial Diseases, Institute of Dentistry, Helsinki University, Central Hospital, Sairaalatie 8, 08 200 LOHJA, Helsinki, Finland; 3 MD, DDS, PhD, Associate Professor, Department of Medicine and Bucofacial Surgery, Complutense University of Madrid, Spain; 4 MD, DDS, PhD, Chairman, Department of Oral and Maxillofacial Diseases, Institute of Dentistry, Helsinki University, Central Hospital, Sairaalatie 8, 08 200 LOHJA, Helsinki, Finland

## Abstract

Objectives: The main objectives are to present the different adverses effects of the immunomodulatory drugs that can impair the quality of life of the immunosupressed patients and study the impact of immunomodualtion on oral diseases. Immunomodulatory drugs have changed the treatment protocols of many diseases where immune functions play a central role, such as rheumatic diseases. Their effect on oral health has not been systematically investigated, however. Study Design: We review current data on the new immunomodulatory drugs from the oral health perspective based on open literature search of the topic. Results: These target specific drugs appear to have less drug interactions than earlier immunomodulating medicines but have nevertheless potential side effects such as activating latent infections. There are some data showing that the new immunomodulatory drugs may also have a role in the treatment of certain oral diseases such as lichen planus or ameliorating symptoms in Sjögren´s syndrome, but the results have not been overly promising. Conclusions: In general, data are sparse of the effect of these new drugs vs. oral diseases and there are no properly powered randomized controlled trials published on this topic.

** Key words:**Immunomodulatory drugs, oral diseases, adverse effects, therapeutic action.

## Introduction

Immunomodulatory drugs modify the response of the immune system by increasing (immunostimulators) or decreasing (immunosuppressives) the production of serum antibodies (1). Immunostimulators are prescribed to enhance the immune response against infectious diseases, tumours, primary or secondary immunodeficiency, and alterations in antibody transfer, among others (2). Immunosuppressive drugs are used to reduce the immune response against transplanted organs and to treat autoimmune diseases such as pemphigus, lupus, or allergies (3,4). In this review article we describe the concept and role of immunomodulation in oral medicine and dentistry with emphasis on new immunomodulatory drugs.

## Material and Methods

The review is based on open PubMed search up to June 2012 using the following key words: immunomodulatory drugs and oral health (17 hits), oral diseases (40 hits), dental (12 hits), lichen planus (4 hits), pemphigus vulgaris (3 hits), pemphigoid (8 hits), erythema multiforme (2 hits), Stevens-Johnson syndrome (2 hits), systemic lupus erythematosus (31 hits), Sjögrens´s syndrome (11 hits), autoimmune disease (426 hits). Relevant articles were then investigated.

This work was made into an investigation Project from Mutua Madrileña adjudicated to Prof. Antonio Bascones-Martinez (ref. AP87102011)

## Results and Discusion

-Mechanisms of action of immunomodulators

**Figure 1:**
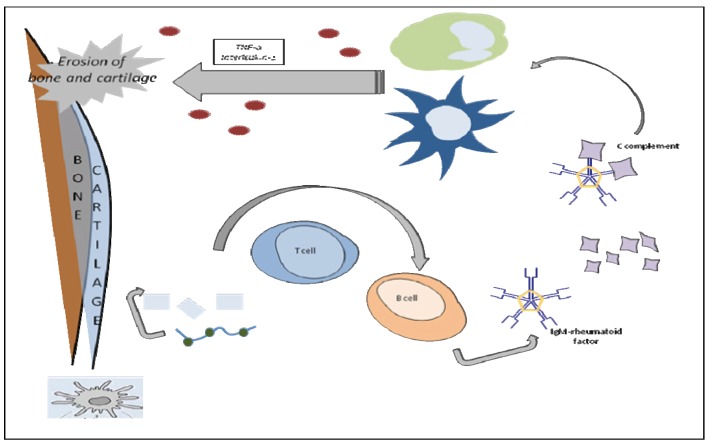
Inflammation in the Rheumatoid Joint. Exogenous antigens are taken up by antigen-presenting cells (APCs). These include: phagocytic cells like dendritic cells and macrophages and, B lymphocytes (B cells). Cytokines are produced by stimulation of polymorphonuclear leukocytes and macrophages.

Immunomodulators act at different levels of the immune system. Therefore different kinds of drugs have been developed that selectively either inhibit or intensify the specific populations and subpopulations of immune responsive cells, i.e. lymphocytes, macrophages, neutrophils, natural killer (NK) cells, and cytotoxic T lymphocytes (CTL). Immunomodulators affect the cells producing soluble mediators such as cytokines (5). Thus, in immunotherapy the immune system is targeted in order to help the healing of a given disease. As an example, the inflammatory processes involved in rheumatoid arthritis are shown in figure 1.

Immunosuppressants inhibit the immune response in organ transplantation and autoimmune diseases, whereas immunostimulants increase the immune response in infections, immunodeficiency (for example AIDS) and cancers. The term immunomodulation is used rather than immunostimulator for a substance that causes measurable alterations in immune function. Their action can be specific or nonspecific.

Specific-action immunomodulators affect the immune system of the cells according to the presence of a particular antigen or immunogen, with selective specificity for immune response. Immunomodulation is selective when the stimulation translates into an immunoreaction to one or several antigens, as in the case of adjuvants or therapeutic vaccines. Immunological adjuvants enhance the effect of vaccines with synthetic antigens, including new-generation antigens. These agents are also used in experimental immunization to obtain polyclonal antiserums and monoclonal antibodies for utilization in vaccines (5).

Non-specific-action immunomodulators are used to stimulate or suppress the immune response, without directing the activity of stimulated cells to a specific antigen. They are divided into three types: type I, acting on normal immune system; type II, acting on immunosuppressed immune system; and type III, acting on functionally normal and immunosuppressed immune system (5).

Autoimmune diseases present with varying symptoms and signs depending on the type of disease and on the individual affected. Thus, for example, skin and joints can be involved in lupus, whereas skin, kidney, and lungs can be involved in other autoimmune diseases. Immunosuppressants are among the most effective drugs also in the treatment of inflammatory bowel diseases. For example, corticosteroids are used in Crohn’s disease to avoid reactivation and post-surgical relapse (6).

-Therapeutic action of different immunomodulators 

 Table 1, Table 2 give the immunostimulating and immunosuppresssant drugs, respectively, and their pharmacological effects. Im-munomodulators are used when the immune system is inadequate to reduce an infection or combating cancer, for example (6). But as can see from the tables, there are a number of different agents with immunomodulatory effects used for a variety of therapeutic purposes. Of the new generation immunomodulators, tumour necrosis factor (TNF) antagonists are specially mentioned here. TNF-α, an inflammatory cytokine released by activated monocytes, macrophages, and T lymphocytes, promotes inflammatory responses that are important in the pathogenesis of rheumatoid arthritis. Patients with rheumatoid arthritis have high concentrations of TNF-α in their synovial fluid (7). Hence the TNF antagonists are widely used in rheumatic diseases.

**Table 1:**
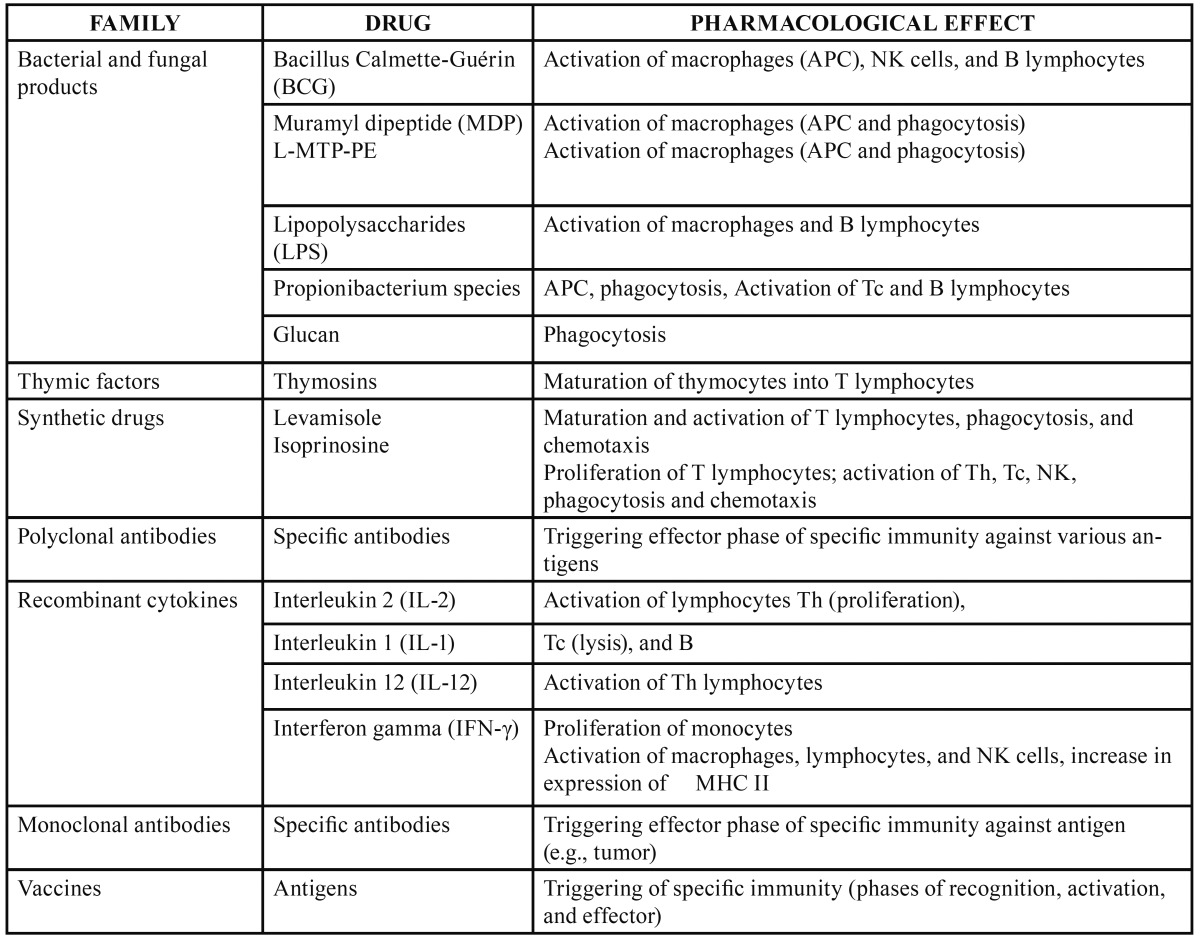
Immunostimulators.

**Table 2:**
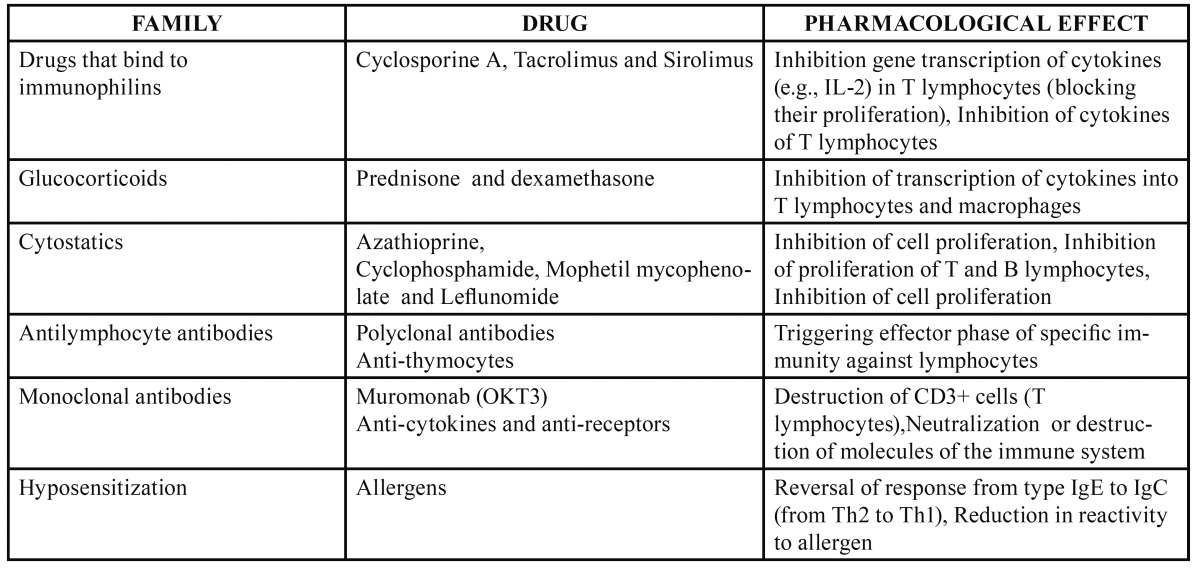
Immunosuppressants.

-Adverse effects

By acting on the immune system, all these drugs may increase the risk of infection. Although usually mild and risk-free, the infections can also be severe, including those caused by opportunistic agents (8). Reactivation of latent tuberculosis is also a known untoward effect particularly reported in connection with the new generation immunomodulatory drugs (8). In general, adverse effects impair the quality of life of the immunosuppressed patient, and pharmacological effects represent the leading cause of death in transplant patients, for example (9). Interestingly, we could not find any literature regarding eventual activation of periodontal disease, for example, in patients taking immunomodulatory drugs. In theory such an untoward development might be possible (10).

Azathioprine and 6-mercaptopurine can cause medullar suppression and a periodical blood count is mandatory for patients receiving these drugs. It is also recommended to take gastric protectors to avoid possible gastric irritation. Other adverse effects include pancreatitis, hepatitis, myalgia, and dizziness, among others. Because of adverse effects azathioprine needs to be withdrawn in 15-30% of patients (11). Adverse effects are observed in 10% of patients taking 50mg/day of azathioprine and can be divided into dose-independent or idiosyncratic effects (rash, fever, alopecia, diarrhoea, and pancreatitis) and dose-dependent or toxic effects (nausea, myelotoxicity and hepatotoxicity) (12,13). Around 5% of patients show elevated transaminase levels (14) and there have been reports of severe bacterial infections, tuberculosis, atypical mycobacterial infection, aspergillosis, histoplasmosis, coccidioidomycosis, listeriosis, Pneumocystis carinii pneumonia, cryptococcal infections, cytomegalovirus, and other infections (8). These infections are common among patients over 65 years old.

Methotrexate, which can be administered intramuscularly or intravenously but also orally, may cause flu-like syndrome, nausea, vomiting, fatigue, diarrhoea, medullar toxicity, and detrimental effects on the lung or liver. The toxicity of methotrexate is dose-dependent and therefore influenced by factors that affect its absorption, distribution, and excretion (15). High doses produce acute and transient elevations of aspartate transferase (AST) and are associated with myelosuppression, mucocutaneous reactions, pneumonitis, and gastrointestinal disturbances (anorexia, nausea and diarrhoea). Prolonged low doses, as in the treatment of psoriasis, rheumatoid arthritis, inflammatory bowel disease, and liver disorders, produce abnormalities ranging from analytical alterations to chronic liver disease, fibrosis, and cirrhosis (15). Methotrexate is contraindicated in women who are pregnant or breast feeding.

Cyclosporine is administered orally or intravenously. Adverse effects include hypertension, nephropathy, convulsions, hyperkalemia, shaking, and hepatitis, among others (11). The most common side effects derive from cyclosporine nephrotoxicity, including hypertension, neurotoxicity, hirsutism, epilepsy, headache, paresthesia, and gingival hyperplasia. In clinical practice, cyclosporine-induced liver damage is characterized by increased serum alkaline phosphatase levels and a mild elevation of aminotransferases and conjugated bilirubin. These biochemical abnormalities usually appear between the second week and third month after initiation of treatment and tend to normalize after reducing the dose (15). Cyclosporine use during pregnancy is not recommended. Mycophenolate and tacrolimus are alternatives to cyclosporine but these drugs may increase the risk of diabetes (16). Reported adverse effects of tacrolimus are local irritation, tingling, burning sensation, altered taste, nausea, headache, and moderate diaorrhea, even when drug absorption is within the therapeutic range of absorption according to urine, blood, and liver analyses; however, tacrolimus hepatotoxicity is relatively uncommon (17).

TNF antagonists are monoclonal antibodies such as infliximab, adalimumab, certolizumab pegol, and golimumab, or circulating receptor fusion protein such as etanercept. The drugs may activate tuberculosis most often due to the reactivation of latent infection and usually within the first two to five months of treatment. Headache, nausea, dizziness, blood glucose changes, epistaxis, infection, and decreased platelets and white blood cells have also been described as side effects of the TNF antagonists. But also lymphoma has been reported in association with TNF antagonists, although a causal relationship is controversial (7).

Finally, corticosteroids widely used for a number of indications need to be mentioned. The most severe acute adverse effect from the use of corticosteroids is adrenal crisis due to their abrupt withdrawal after prolonged administration. Other reported side effects are hypertension, hypercholesterolemia, glucose intolerance, insomnia, emotional lability, psychotic disorders, cataract, osteopenia with vertebral compression, diabetes, and cosmetic changes, such as “moon face” (Cushing syndrome), “buffalo hump”, acne and hirsutism. These side effects appear in 80% of patients after 2 years of treatment and resolve on withdrawal of the corticosteroid. With regard to effects on the liver, high doses of glucocorticoids can produce hepatic steatosis by promoting the mobilization and redistribution of fat, increasing plasma free fatty acids by inhibiting the esterification of fatty acids in the liver (18).

-Drug interactions

A drug interaction is the modification of the pharmacodynamic and/or the pharmacokinetics of a drug as a result of the joint processing of other medications or foods or habits such as snuff use or frequent alcohol consumption. For example, leflunomide may increase the anticoagulant activity of warfarin (19,20). The spectrum of drug interactions ranges widely from those without any clinical relevance to those that produce a severe adverse reaction in the patient.

In general, drugs used by dental practitioner, such as antibiotics, pain killers and local anaesthetics, are well tolerated with the new biological medicines. Most of the drugs undergo some degree of biotransformation before elimination. It has been well established that the biological target for the vast majority of these drug metabolic interactions is in the cytochrome P450 system (CYP) (21). Schmitt et al. have shown that tocilizumab, an interleukin-6 receptor inhibitor, may reverse IL-6-induced suppression of CYP3A4 activity and thus “normalizes” CYP3A4 activity to a level similar to that in healthy persons (22). This finding showed the importance of caution with patients taking tocilizumab and simvastatin, and thus with any other CYP3A4 metabolized drugs. Special interest to dental treatment are the CYP3A4 substrates, such as the local anaesthetic lidocaine, and the popular anxiolytics midazolam and diazepam, and CYP3A4 inhibitors, such as erythromycin and clarithtromycin and macrolide antibiotics in general, and azole antifungal drugs in particular (21,23). Apart from the increased drug concentrations observed with simultaneous use of the certain antifungal agents and immunomodulating drugs, however, there is no evidence for interactions with the drugs commonly used in the dental practice and the novel biologic agents discussed here.

-Impact of immonumodulation on oral diseases

Autoimmune diseases frequently involve the oral mucosa which often is the first site of manifestation (24). A detailed clinical examination of the oral mucosa of an asymptomatic patient can be the best opportunity for the early diagnosis and treatment of these autoimmune diseases, allowing control over their spread to the skin and/or other body organs (25).  Table 3 lists the im-portant autoimmune diseases with oral manifestations.

**Table 3:**
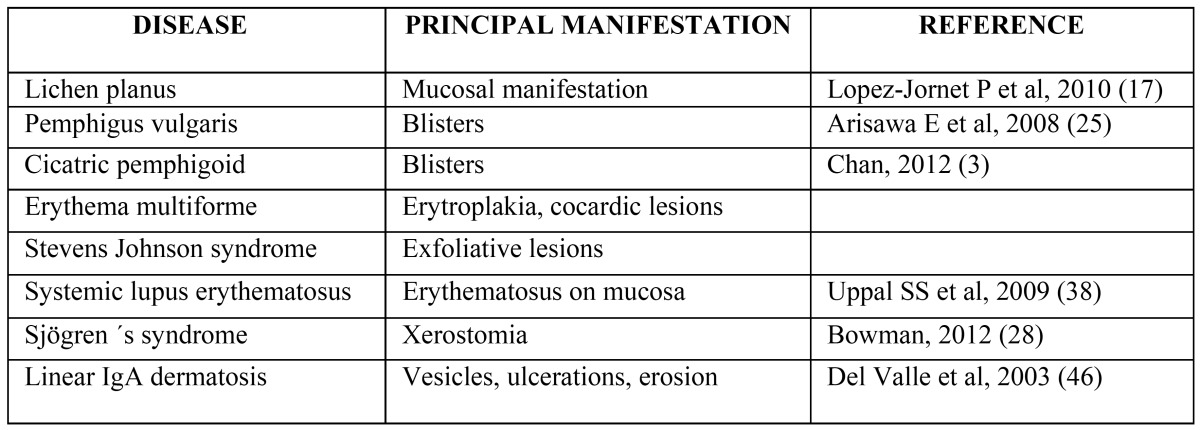
Oral manifestations autoimmune diseases.

Novel therapies based on pathogenesis 

Interest in B-cell–targeted therapies has increased worldwide following recent convincing evidence that innate immunity, most notably mediated by INF signalling, plays a role in initial B-cell activation. Numerous drugs under current evaluation, including epratuzumab, a monoclonal antibody directed at the CD22 B-cell surface antigen, which may preferentially target auto reactive B cells, target the B lymphocyte pathogenic axis (see Fig. 1). Baminercept, a lymphocytotoxin-beta receptor fusion protein, which along with BAFF supports the formation of germinal centers within salivary glands, is another molecule of interest for autoimmune diseases (26).

Particular promise has been shown by belimumab, a monoclonal antibody that specifically targets the BAFF receptor and may disrupt the cycle of B-cell activation and antibody production. Belimumab appears to be effective for systemic lupus erythematosus (SLE) and is undergoing early stage development for SS (27).

Potential new cytokine therapeutic targets were recently suggested by data to implicate proinflammatory Th17 cells in SS. IL-17 and IL-23, as well as the related pro-inflammatory cytokines IL-12 and IL-6, are prominently expressed in SS salivary gland tissue (26).

Rituximab was the first B-cell–targeting therapy to be evaluated in SS. Rituximab is a mouse-human (chimeric) antibody directed against the CD20 cell surface antigen present on B cells. It was introduced as treatment for primary lymphoma and results in the depletion of circulating B cells. The usefulness of rituximab to treat lymphoma and knowledge of the role played by B-cell hyperactivity in the systemic manifestations of SS led to its proposal for therapeutic application in SS some years ago (28). The use of B-cell–depleting therapies in SS is supported by evidence that rituximab treatment depletes B cells in parotid gland tissue and in the peripheral blood, as well as it restores normal T-cell regulatory function, reduces glandular inflammation, and improves the function and regression of lymphoepithelial lesions that predispose to the development of lymphoma (29).

Rituximab was found to improve subjective sicca symptoms, fatigue, and quality-of-life (29). Two small randomized double-blind controlled studies have demonstrated its efficacy and safety in SS (30,31). The evidence suggests that rituximab is effective for extra-glandular manifestations of SS (29-31).

In contrast to B-cell inhibitor, rituximab as an anti-TNG agent has not shown any evidence of efficacy in SS treatment (32). Similar results were detected with another anti-TNF-alpha antibody (etanercept, infliximab. However, it seems that the new immonumodulatory drugs in general have shown disappointing results in treatment of SS as reviewed by Carsons (4). The author stresses the need for properly powered and controlled studies on the topic.

Etanercept, efalizumab and alefacept were used in treatment of oral lichen planus (OLP). Etanercept inhibits binding of TNF-alpha to cell surface TNF receptors and thus prevents TNF-mediated cellular responses. It has been shown to relief OLP symptoms in two weeks after commencement of therapy and clinical improvement in four weeks (33). Efalizumab and alefacept, both T-cell inhibitors, have also been reported to be successful in the treatment of OLP (34). Reason for the efficacy of these agents is thought to be due to increased activation of T-cells and proliferation. In contrast to this finding, Asarch et al. demonstrated lichen planus-like eruptions after infliximab and adalimumab therapy for psoriasis (35). They suggested that TNF-alpha inhibition may precipitate lichenoid reactions through disruption of delicate balance between TNF-alpha and interferon-alpha in susceptible patients. Similar result was found with a patient with Crohn’s disease after certolizumab pegol treatment (36). Hence the issue remains controversial and more studies are called for further evidence if or not these drugs have a role in the treatment of lichen planus.

In SLE, TNF is a proinflammatory and regulatory cytokine with divergent effector on the immune system. Thus, TNF inhibition by infliximab and etanercept are of interest in SLE treatment. It has been shown that in a short-term therapy, infliximab is effective and relatively safe in SLE treatment but in the long-term therapy infliximab, however, was associated with severe infections and potentially also lymphomas are of concern if therapy is continued (37,38).

B-cell activation and autoantibodies are characterized in SLE. Thus, rituximab as an anti-CD20 antibody is of interest in evaluating its effectiveness in SLE treatment. Results have indicated that rituximab was indeed effective in SLE, and clinical responses were supported by close correlation with B cell numbers (39,40). Moreover, SLE is known as a highly heterogeneous disease. This might explain why not all patient show response with rituximab treatment while with abatacept, a T cell inhibitor, the results were promising (41-43). However, rituximab is considered as the first-choice biological agent in patients with SLE (42).

Interleukin-6 (IL-6) is a key proinflammatory cytokine and the serum levels of IL-6 are elevated with SLE patients (44). Inhibition of IL-6 by tocilizumab has shown promising responses in clinical and serologic manifestations of lupus activity in patients with SLE (45). Nevertheless, Illei et al. found developing dose-related neutropenia and high rates of infections in almost all patients in their study with tocilizumab treatment (45,46). Thus, further studies are needed with IL-6 receptor inhibition and SLE. The future use of these drugs in oral medicine remains open (47).  Table 4 summarizes the biological drugs used in the treatment of autoimmune diseases.

**Table 4:**
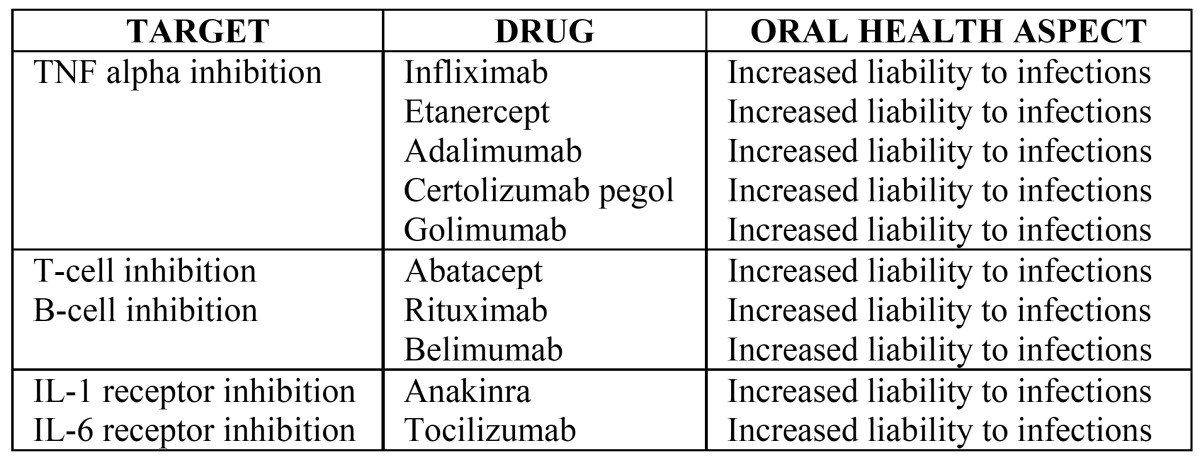
Biologic agents in the treatment of autoimmune diseases.

## Conclusion

We have briefly reviewed current knowledge about the new immunomodulatory drugs from the oral health perspective. The data available show that these drugs appear safe as regards possible interactions with commonly used medicines in dental practice, such as antibiotics, pain killers and local anaesthetics. However, drug interactions may occur with some antifungal agents. The interpretation and evaluation of potential adverse effects and drug interactions of patients has been described as a daily challenge for dentists in general and alertness is called for with the introduction of new drugs and drug categories. Another area of interest here is the use of the new immunomodulatory drugs in treatment of autoimmune diseases with oral manifestations. These include diseases such as Sjögren´s syndrome and oral lichen planus. So far, however, data are sparse to give any clinical guidelines and properly powered randomized controlled trials are needed for scientific evidence.
